# Culture Ordering for Patients with New-onset Fever: A Survey of Pediatric Intensive Care Unit Clinician Practices

**DOI:** 10.1097/pq9.0000000000000463

**Published:** 2021-08-26

**Authors:** Lauren D. Booth, Anna C. Sick-Samuels, Aaron M. Milstone, James C. Fackler, Lindsey K. Gnazzo, David C. Stockwell

**Affiliations:** From the * Department of Anesthesiology and Critical Care Medicine, Johns Hopkins Hospital, Baltimore, Md.; † Department of Pediatrics, Johns Hopkins School of Medicine, Baltimore, Md.; ‡ Department of Hospital Epidemiology and Infection Control, Johns Hopkins Hospital, Baltimore, Md.; § Johns Hopkins University, School of Nursing, Baltimore, Md.

## Abstract

**Introduction:**

Accurate assessment of infection in critically ill patients is vital to their care. Both indiscretion and under-utilization of diagnostic microbiology testing can contribute to inappropriate antibiotic administration or delays in diagnosis. However, indiscretion in diagnostic microbiology cultures may also lead to unnecessary tests that, if false-positive, would incur additional costs and unhelpful evaluations. This quality improvement project objective was to assess pediatric intensive care unit (PICU) clinicians’ attitudes and practices around the microbiology work-up for patients with new-onset fever.

**Methods:**

We developed and conducted a self-administered electronic survey of PICU clinicians at a single institution. The survey included 7 common clinical vignettes of PICU patients with new-onset fever and asked participants whether they would obtain central line blood cultures, peripheral blood cultures, respiratory aspirate cultures, cerebrospinal fluid cultures, urine cultures, and/or urinalyses.

**Results:**

Forty-seven of 54 clinicians (87%) completed the survey. Diagnostic specimen ordering practices were notably heterogeneous. Respondents unanimously favored a decision-support algorithm to guide culture specimen ordering practices for PICU patients with fever (100%, N = 47). A majority (91.5%, N = 43) indicated that a decision-support algorithm would be a means to align PICU and consulting care teams when ordering culture specimens for patients with fever.

**Conclusion:**

This survey revealed variability of diagnostic specimen ordering practices for patients with new fever, supporting an opportunity to standardize practices. Clinicians favored a decision-support tool and thought that it would help align patient management between clinical team members. The results will be used to inform future diagnostic stewardship efforts.

## Abstract 2

Supplemental Digital Content is available in the text.

## INTRODUCTION

Body fluids, such as blood, respiratory secretions, and urine, are commonly cultured in the pediatric intensive care unit (PICU) to assess for infections such as sepsis, ventilator-associated pneumonia, or urinary tract infections. However, indiscretion in diagnostic microbiological specimen ordering may lead to undesirable outcomes, such as false-positive results and then inappropriate antibiotic use.^1^ Diagnostic stewardship provides an upstream strategy to mitigate downstream complications while also permitting safe and effective care.^2–5^ For example, blood cultures drawn from a central line can be more likely than peripheral blood cultures to grow colonized flora, leading to a false-positive impression of bloodstream infection.^6–8^ Similarly, expectorated sputum culture samples from children typically reflect the oral flora bacteria rather than pathogens from the lower respiratory tract.^9^ Judicious use of microbiology testing has been successfully demonstrated for blood, urine, and *Clostridium difficile* testing with associated reductions in avoidable antibiotic treatment, adverse events, and healthcare costs.^1,10–13^

Fever is a common trigger for additional work-up of patients in the PICU. Our PICU previously undertook separate diagnostic stewardship initiatives that focused on blood^14^ and endotracheal aspirate cultures.^15^ The continued impact of these diagnostic stewardship initiatives on clinical practice remains unknown. The initial blood culture clinical decision-support tool demonstrated a decrease in the number of blood cultures per 100 patient-days without an increase in suspected sepsis or a change in mortality or hospital readmission rates.^14^ The team assessed adherence to the blood culture algorithm over the initial months after its introduction; however, we do not know how practices may have diverged over time.

No national consensus provides specific recommendations for culture ordering practices within the PICU setting, and blood culture ordering practices vary considerably across the country.^16^ Additionally, we have not made efforts to assess or standardize urine or cerebrospinal fluid (CSF) cultures in our unit. Therefore, we hypothesized that the work-up of febrile patients would be variable. This project’s objective was to assess PICU clinicians’ attitudes and practices around the common diagnostic microbiology specimens ordered for PICU patients with new-onset fever.

## METHODS

### Setting

A multidisciplinary workgroup of PICU and pediatric infectious disease clinicians created a self-administered electronic survey to ascertain current culture practices among PICU clinicians, including attendings, fellows, and frontline clinicians (nurse practitioners and hospitalists) for PICU patients with new-onset fever. The survey was conducted at the Johns Hopkins Children’s Center PICU, a 40-bed facility with medical, surgical, and cardiac patients. We defined a new fever as a fever ≥48 hours from the prior fever. We categorized immunocompetent fever as 38.2 °C for 2 measurements 1 hour apart, 38.5 °C for 1 measurement, or <36 °C for 2 measurements 1 hour apart. We categorized immunocompromised fever as ≥38°C for 2 measurements 1 hour apart or >38.3 °C for 1 measurement.

### Survey

The survey included questions concerning clinician role, work experience, and attitudes and perceptions about microbiology testing for PICU patients with a new fever. The survey presented 7 clinical vignettes and asked clinicians to select which diagnostic samples they would or would not obtain. The 7 vignettes addressed various common clinical scenarios for patients with new fever (see Table 1 and **Supplemental Digital Content 1,** http://links.lww.com/PQ9/A301). The diagnostic sample options included central line blood cultures, peripheral blood cultures, respiratory aspirate cultures, urine cultures, urinalyses, and CSF cultures. Each vignette also included a free-text option to allow clinicians to write in other diagnostic tests that they would obtain. To optimize chances that responders read the full question stem, we aimed to keep the clinical vignettes brief while still communicating the core of the scenario at the expense of including clinical detail that some clinicians may factor into their decision-making. The vignettes were internally piloted within the authorship group and then piloted by a small group of PICU clinicians who were not within the project workgroup to ensure comprehension outside of the study team.

**Table 1.** Description of Clinical Scenarios

Scenario	Description
1	Patient with a central line thrombus
2	Patient from scenario 1 with recurring fever
3	Patient with a postoperative fever
4	Patient with new-onset ventilator-associated pneumonia
5	Patient with neonatal sepsis
6	Patient with sedation withdrawal
7	Patient with fever and neutropenia

The survey was administered via the Qualtrics Core XM (Provo, Utah, 2020) program and was sent to the PICU attending, fellow, and frontline clinician groups via email from November 20, 2019, through January 3, 2020. We intentionally did not administer the survey to resident trainees because residents often rotate through the PICU. They do not make decisions regarding culture orders. We were interested in the local culture ordering practices of our clinicians. Reminder emails to complete the survey were sent once a week. Participation in the survey was voluntary, anonymous, and without compensation. The Johns Hopkins Institutional Review Board acknowledged this survey as part of a quality improvement project.

### Data and Analysis

Responses were summarized as descriptive statistics of frequencies and percentages. Although clinicians were originally able to respond to the clinical scenarios as “yes,” “no,” or “unsure,” “unsure” selections were rare. Therefore, we combined “unsure” with “no” responses, creating a binary response. We assessed responses for deviation from the existing decision-support algorithms or standards of care for blood cultures,^14^ respiratory aspirate cultures,^15^ immunocompromised patients,^17^ and neonatal sepsis evaluations.^18,19^ The free-text responses were reviewed for other noninfectious and infectious sources of fever.

Three levels of consensus were assigned and displayed as a heatmap based on the proportion of clinicians who would obtain a diagnostic specimen for the 7 clinical scenarios. We assigned “consensus” (green) when either most clinicians stated that they would obtain a culture (85%–100% would) or most clinicians would not obtain a culture (0%–16% would). Similarly, we assigned “moderate consensus” (yellow) when either 68%–84% or 17%–33% of clinicians would obtain a culture. Last, we assigned “no consensus” (red) when 34%–67% of clinicians would obtain a specimen.

## RESULTS

Overall, 47 of 54 invited participants responded to the survey, yielding an 87% response rate. Of the invited participants, 21 of 23 attendings responded (91.3%), 12 of 15 fellows responded (80%), and 14 of 16 frontline clinicians responded (87.5%). Thus, the participant group’s breakdown was 44.7% attending physicians, 29.8% nurse practitioners/hospitalists, and 25.5% fellows. Experience level was equally distributed within the group, which included 12 fellows (25.5%), 12 respondents with less than 5 years of posttraining experience (25.5%), 11 respondents with 5–10 years of posttraining experience (23.4%), and 12 respondents with more than 10 years of posttraining experience (25.5%).

All respondents (N = 47, 100%) favored a decision-support algorithm for obtaining a culture specimen in PICU patients with fever. Similarly, 91.5% (N = 43) of participants responded that it would be helpful to have a decision-support algorithm to align PICU care teams and/or consulting care teams. Fifty-seven percent (N = 27) of respondents perceived themselves as not well-informed regarding optimal microbiology culturing practices. Simultaneously, two-thirds of participants (N = 31, 66%) felt confident in ordering microbiology cultures for PICU patients with fever. Approximately, half of the participants sometimes reported disagreeing with another care team member about which culture to obtain (1–2× per week; N = 23, 49%). The other half reported that disagreements rarely occurred (1–2× per month; N = 23, 49%). Clinicians also expressed a preference for an algorithm over other types of potential educational material for microbiology diagnostic specimen collection (44.3%, N = 43; Table 2).

**Table 2.** Perceptions of Diagnostic Culture Ordering Practices and Current Diagnostic Culture Ordering Resources (N = 47)

Question	N (%)
Would you find a decision-support algorithm helpful when deciding to obtain culture specimens in PICU patients with fever?	
Yes	47 (100)
No	0 (0)
Do you think a decision-support algorithm would help align the PICU care team and/or consulting care teams regarding culture specimen collection?	
Yes	43 (91.5)
No	4 (8.5)
Do you consider yourself well-informed regarding best practices for obtaining microbiology cultures in PICU patients with fever?	
Yes	20 (42.6)
No	27 (57.4)
Do you think there is variability in practice between different clinicians in our PICU regarding when to obtain microbiology cultures?	
Yes	45 (95.7)
No	2 (4.3)
How often have you disagreed with another member of the patient’s care team regarding which microbiology cultures should or should not be obtained?	
Frequently (almost every shift)	1 (2.1)
Sometimes (1–2× weekly)	23 (48.9)
Rarely (1–2× monthly)	23 (48.9)
Never	0 (0)
How confident do you feel in selecting which types of microbiology cultures to obtain in response to PICU patients with fever?	
Highly confident	1 (2)
Confident	31 (66)
Neutral	13 (27.7)
Unsure	2 (4.3)
Highly unsure	0 (0.0)
What resources have you used to help inform your decisions regarding culture specimen collection with fever? N = 91	
Textbook	16 (17.6)
UpToDate	22 (24.2)
Google Scholar	2 (2.2)
JHH resource	29 (31.9)
Other online resource	4 (4.4)
PubMed	16 (17.6)
I do not use resources	2 (2.2)
What other types of education material would you find helpful regarding culture specimen collection? N = 97	
Lecture	10 (10.3)
Online learning module	17 (17.5)
Algorithm	43 (44.3)
Electronic resource	27 (27.8)
I am not interested in more education	0 (0)

JHH, Johns Hopkins Hospital.

**Fig. 1.**
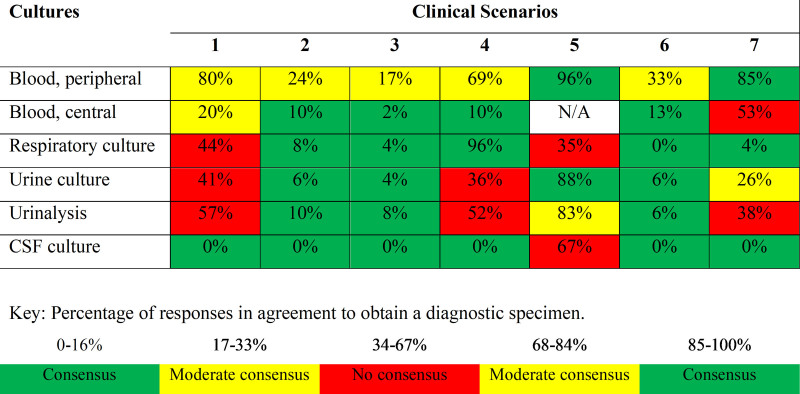
Percentage of participants who would obtain a diagnostic sample across seven clinical scenarios with the degree of consensus between participants shown as a heatmap. CSF, cerebrospinal fluid; N/A, not applicable.

According to the heatmap created from vignette survey results (Fig. 1), variability was highest for peripheral blood cultures (5 of the 7 clinical scenarios had moderate consensus) and urinalysis (3 of the 7 scenarios had no consensus, and 1 of 7 had moderate consensus). Two of the 7 scenarios had no consensus for urine culture specimens, and one of the 7 had moderate consensus. CSF cultures had the most consensus among all diagnostic specimens, as 6 of the 7 scenarios showed consensus. There was no consensus for CSF cultures among responses for the patient with neonatal sepsis (scenario 5). When we considered each clinical scenario, four of the seven had no consensus regarding the culture of 2 or more specimen types: central line thrombus (scenario 1), new-onset ventilator-associated pneumonia (scenario 4), neonatal sepsis (scenario 5), and fever with neutropenia (scenario 7).

## DISCUSSION

Our survey revealed variability in diagnostic ordering practices among clinicians for PICU patients with new-onset fever despite 2 prior quality improvement projects to standardize blood^14^ and respiratory^15^ culture ordering practices. To date, we have not implemented a quality improvement project to standardize urine or CSF fluid collection in PICU patients with fever. Therefore, it is not surprising that consensus was the lowest for these diagnostic specimens.

Interestingly, the consensus was highest for obtaining peripheral blood cultures in patients with neonatal sepsis (scenario 5) and fever with neutropenia (scenario 7). For the remaining 5 scenarios, the consensus was only moderate among the responses to obtain a peripheral blood culture despite an existing blood culture decision-support tool. These findings led us to infer that the clinicians do not utilize the current clinical decision-making algorithm for blood culture ordering, the clinicians disagree with the algorithm, or the clinicians did not consider the current algorithm while completing the survey. The results may also represent a deviation from the existing blood culture decision support tool introduced in 2014.^14^ In this period, the pool of fellow trainees has completely turned over, and new frontline clinicians have joined our PICU. This rate of change may highlight the need for biannual standardized education for our existing clinical support tools. When we compared our results against our current respiratory culture algorithm, which was introduced in 2018,^15^ several clinicians selected a respiratory culture for a patient without an endotracheal or tracheostomy tube (neonatal sepsis scenario 5). Although expectorated samples may be helpful in the adult population, pediatric patients may not purposefully expectorate. Therefore, such samples reflect oral flora only and should be avoided.^9^ The survey underscored the need for further education and reinforcement of our current blood and respiratory culture ordering practice recommendations. This survey also emphasized the need for a structured Plan-Do-Study-Act (PDSA) cycle for our PICU quality improvement efforts.^20^ Although both of our previously implemented clinical support devices led to an initial decrease in uninformative culture ordering practices,^14,15^ the necessary iterative PDSA cycle was not repeated for continued unit engagement and practice reflection. These decision-support tools are also standalone resources available in 2 different electronic forms, contributing to clinician lack of compliance. Our goal in future algorithm iterations is to provide clinicians with a comprehensive guide to fever evaluation within one document. We would like to monitor adherence with the recommendations and will consider integrating the tools into the electronic medical record to support sustained practice change.

Although standardization is an essential component of providing safe and effective care, we recognize that homogeneity is not synonymous with best practice. For example, there was no consensus for CSF culture in the neonatal sepsis vignette (scenario 5), but this was the one clinical scenario for which a CSF culture was warranted.^18,19^ Likewise, there was no consensus for obtaining a central line blood culture in the vignette describing new-onset fever with neutropenia (scenario 7) in an immunocompromised patient with a central line.^17^ Again, this scenario underscored that consensus is beneficial only when it aligns with best practice. It will be important to stress these real-life evaluations of neonatal sepsis and the immunocompromised patient in future decision-making tools and quality improvement efforts in our unit. Curiously, the consensus was lowest among providers with more than 10 years of experience. This group also expressed the highest rate of feeling uninformed about best microbiology ordering practices for PICU patients with fever, a circumstance that may account for the lack of consensus. An additional study may be warranted to better understand the variability among the most experienced clinicians both within our institution and across institutions.

The overall heterogeneity of responses highlighted inconsistency among our PICU clinicians concerning diagnostic specimen ordering practices and presents an opportunity to standardize clinical practice. A clinical support tool, such as an algorithm, is a diagnostic stewardship device that improves medical management if properly designed and used. A clinical support tool does not replace clinicians’ expertise; instead, these tools support adherence with evidence-based practice^21^ and reduce practice variability within similar clinical scenarios for more consistent medical care. These support tools also mitigate cognitive biases that clinicians carry into their clinical decision-making process.^22,23^ For example, 3 of the 7 scenarios were focused on noninfectious sources of fever: central line thrombus (scenario 1), postoperative fever (scenario 3), and sedation withdrawal (scenario 6). We suspect that clinicians still obtained microbiology studies given the cognitive bias of fever within the stem. As clinicians, we need to recognize cognitive biases and systematically evaluate patients against known evidence-based standards of care. With excessive testing from cognitive biases, patients may receive prolonged antibiotic courses, be misdiagnosed, or undergo unnecessary diagnostic evaluations based on false-positive results.^2–5^ These patient-level outcomes align well with diagnostic stewardship goals: improving patient management by reducing low-yield cultures and improving diagnostic accuracy.^1^ In future clinical decision-support tool iterations, we plan to evaluate other patient-level outcomes, such as adverse outcomes from antibiotic use, central line complications, length of stay, mortality, and readmission rates. We recognize that one-fifth of pediatric patients have adverse outcomes from antibiotic administration,^24^ such as acute kidney injury or *Clostridium difficile* infections, and hypothesize that any reduction in culture ordering practice and subsequent antibiotic administration will improve patient care. With the integration of neonatal sepsis, fever and neutropenia, and meningitis into our future clinical decision-support tool, we also hypothesize earlier identification and treatment of these conditions with improved length of stay and functional outcomes. Implementation of any support tool should be accompanied by monitoring clinical outcomes and potential unintended consequences.^14^

This project had several limitations. Based on the 87% response rate, we have confidence that the survey sample was a sufficient proportion of clinicians to reflect the general practices of those working in this unit. Nonetheless, because this was a single-center study, the results may not be generalizable to clinicians from other PICUs or settings. More studies are needed to assess heterogeneity in clinical practice at other institutions. Therefore, other facilities could consider using this survey to assess their internal procedures. We can speculate that if practice variability was prominent within one unit, it might be present across institutions. We also recognize that the responses to a vignette-style survey may not reflect the complexity of decision-making for real-life patients. The clinical scenario stems may not contain all of the information that individual providers request for decision-making. The scenarios were designed to be common clinical encounters with basic laboratory and pertinent clinical assessment findings. Therefore, we expected the survey responses to reflect true heterogeneity among clinicians’ approaches to the same clinical situation. To optimize the response rate, we did not include all possible clinical scenarios; hence, not all situations were captured by the survey. We also recognize that the survey language may reflect local practice patterns or phrasing. Therefore, vignettes should be reassessed for internal validity at other institutions.

## CONCLUSIONS

This survey revealed heterogeneity of diagnostic specimen ordering practices among PICU clinicians evaluating patients with new-onset fever. Clinicians were unanimously in favor of a decision-support tool and thought it would help align patient management between clinical team members. Our next steps will involve integrating our current blood and respiratory culture algorithms into one tool with the addition of CSF studies, urine studies, and other infectious and noninfectious sources of fever as a means to promote diagnostic stewardship and mitigate cognitive biases.

## DISCLOSURE

The authors have no financial interest to declare in relation to the content of this article.

## ACKNOWLEDGMENTS

We would like to thank the individuals who helped review and pilot the survey: Dr. Tracie Lin, Pediatric Cardiology Fellow, Penn State Children’s Hospital (Hershey, Pa.) and Dr. Elliot Melendez, PICU Attending, Johns Hopkins All Children’s Hospital (St. Petersburg, Fla.).

## Supplementary Material


