# Supplementary material for: Culture Ordering for Patients with New-onset Fever: A Survey of Pediatric Intensive Care Unit Clinician Practices
Source: Pediatr Qual Saf. 2021 Aug 26;6(5):e463. doi: 10.1097/pq9.0000000000000463 (PMC8389917; doi:10.1097/pq9.0000000000000463)
Supplement: Supplementary file 1 [file pqs-6-e463-s001.pdf]

Culture ordering for patients with new-onset fever: a survey of pediatric intensive care unit clinician practices

First Author: Lauren D. Booth, MSN, CRNP*
*Department of Anesthesiology and Critical Care Medicine, Johns Hopkins Hospital, Baltimore, MD

Supplement 1

Scenario 1

You are providing care for a 5-month-old previously healthy female admitted to the pediatric intensive care unit for respiratory distress. The patient was diagnosed with RSV bronchiolitis on hospital day 1 and intubated on hospital day 4. It is currently hospital day 6 and the nurse notifies you that the patient has a new fever to 38.8°C. Her other vital signs are unremarkable aside from mild tachycardia. The patient continues to have the same quantity of moderate, thick yellow secretions. The morning chest x-ray demonstrated peri-bronchial cuffing without focal consolidation.

The patient's labs are unremarkable. For access, the patient has a double lumen central line with no blood return that flushes well and 2 peripheral IVs. The patient is not currently on antibiotics. No culture specimens have been drawn this admission.

Will you obtain the following culture specimens?

Scenario 2

You are still caring for the patient in scenario 1—a 5-month-old previously healthy female with RSV bronchiolitis intubated on hospital day 4 who developed a new fever on hospital day 5. It is now hospital day 6. Yesterday, you decided to obtain a peripheral blood culture and a vascular ultrasound to assess for a thrombus around the double lumen central line that did not have blood return. The ultrasound revealed an occlusive thrombus. The patient continues to be intubated with no other changes in her clinical status. Her vital signs remain unremarkable aside from another fever to 38.5°C. There is no change in her respiratory secretions or morning chest x-ray findings (peri-bronchial cuffing without focal consolidation).

The patient is not on antibiotics. The peripheral blood culture from yesterday is no growth to date.

Will you obtain the following culture specimens?

Scenario 3

You are providing care for a 6-year-old male admitted to the pediatric intensive care unit after a mandibular distraction with the general surgical team. The patient has an extended past medical history, including Pierre Robin and tracheostomy dependence with trach collar during the day and CPAP 5 cm H20 at 21% overnight.

It is now post-op day 1 and the bedside nurse notifies you that the patient is febrile to 38.5°C. He remains hemodynamically stable. On your morning assessment, he is on his trach collar 21% with clear secretions without hypoxia. He is comfortable on his low dose dilaudid and ketamine infusions. You review the patient's labs, which are unremarkable except for anemia (Hgb 9) and leukocytosis (WBC 17). For access, the patient has a double lumen internal jugular central line, 2 peripheral IVs, and a peripheral arterial line. A urinary catheter was removed last night. The patient is currently receiving peri-op Ancef for 24 hours. There are no culture specimens on this admission.

Will you obtain the following culture specimens?

Scenario 4

You are providing care for a 2-year-old male admitted to the pediatric intensive care unit for respiratory failure in the setting of status epilepticus. The patient missed several doses of his anti-epileptic medication at home and then presented to an outside hospital in status epilepticus. He was intubated given his inability to protect his airway and transferred to your hospital. It is currently hospital day 4 and the patient remains intubated given the need to escalate his anti-epileptic agents. The overnight team escalated his PEEP on evening rounds due to persistent hypoxia.

During your morning assessment, the bedside nurse notifies you that the patient is febrile to 38.8°C with more respiratory secretions in comparison to yesterday. His other vital signs are

significant for mild tachycardia. For access, he has a double lumen femoral line, 1 peripheral IV, a peripheral arterial line, and a urinary catheter to straight drain for urinary retention. You review the patient's labs, which are significant for anemia (Hgb 10), leukocytosis (WBC 14.2), and thrombocytosis (432). A chest x-ray demonstrates a new retrocardiac opacification.

The patient is not currently on an antibiotic regimen. The blood, urine, and CSF cultures from admission are no growth to date. The CSF specimen was not concerning for infection. The respiratory viral panel from admission is negative.

Will you obtain the following culture specimens?

Scenario 5

You are providing care for an 18-day-old male with respiratory distress. He was initially transferred from an outside hospital with mild respiratory distress secondary to rhinovirus. He required frequent nasopharyngeal suctioning for respiratory secretions and was stable on high-flow nasal cannula 6L 40% until last night. The overnight medical team increased his respiratory support given his worsening work of breathing and hypoxia.

On your morning assessment, the infant is lethargic, tachypneic with moderate work of breathing on high-flow nasal cannula 10L, 100% with pulse ox 91%. He is tachycardic (HR 195) and hypotensive (MAP 33) with a rectal temperature of 38.3°C. The patient has 1 peripheral IV for access. There are no recent labs on the patient. He is not currently on antibiotics.

Will you obtain the following culture specimens?

Scenario 6

You are providing care for a 12-year-old female admitted to the pediatric intensive care unit for septic shock and a prolonged intubation course on sedating and paralyzing infusions. She was extubated to BiPAP several days ago and now is on high-flow nasal cannula with good effect. A peripherally inserted central catheter line was placed 10 days ago for antibiotics (completed 14-day course of antibiotics) and a prolonged sedation wean. The precedex infusion was weaned yesterday.

During your morning assessment, the bedside nurse notifies you that the patient is febrile to 38.5°C. The patient appears agitated in the bed with mild tachycardia and hypertension. She continues on her nasoduodenal feeds with new onset loose stools and retching. The patient's labs are unremarkable. The patient is not currently on an antibiotic regimen. The most recent blood and respiratory cultures are negative from hospital day 6. Today is hospital day 16.

Will you obtain the following culture specimens?

Scenario 7

You are providing care for an 11-year-old male with relapsed ALL undergoing induction therapy. He was transferred to the pediatric intensive care unit several days ago for respiratory distress in the setting of fever with neutropenia. He has been on stable high-flow nasal settings since admission.

The bedside nurse notifies you that the patient has a new onset fever this morning to 38.5°C. He is tachycardic with HR 150. Of note, the patient has new onset diarrhea. For access, the patient has a double lumen Hickman central line and 1 peripheral IV. The patient is currently on cefepime given his history of fever 5 days ago alongside of his neutropenia. He remains neutropenic. His blood cultures remain no growth to date.

Will you obtain the following culture specimens?
